# Use of theory to plan or evaluate guideline implementation among physicians: a scoping review

**DOI:** 10.1186/s13012-017-0557-0

**Published:** 2017-02-27

**Authors:** Laurel Liang, Susanne Bernhardsson, Robin W. M. Vernooij, Melissa J. Armstrong, André Bussières, Melissa C. Brouwers, Anna R. Gagliardi, Members of the Guidelines International Network Implementation Working Group, Samia Alhabib, Margot Fleuren, Margie Fortino, Danielle Mazza, Niamh O’Rourke, Melina Willson

**Affiliations:** 1 University of Toronto, Toronto, Canada; 2 Närhälsan Research and Development Centre for Primary Health Care, Region Västra Götaland, Göteborg, Sweden; 3 Netherlands Comprehensive Cancer Organisation, Utrecht, The Netherlands; 4 University of Florida College of Medicine, Gainesville, Florida USA; 5 McGill University, Montreal, Canada; 6 McMaster University, Hamilton, Canada; 7 University Health Network, Toronto, Canada

**Keywords:** Guidelines, Implementation, Interventions, Theory, Evaluation, Effectiveness, Physicians

## Abstract

**Background:**

Guidelines support health care decision-making and high quality care and outcomes. However, their implementation is sub-optimal. Theory-informed, tailored implementation is associated with guideline use. Few guideline implementation studies published up to 1998 employed theory. This study aimed to describe if and how theory is now used to plan or evaluate guideline implementation among physicians.

**Methods:**

A scoping review was conducted. MEDLINE, EMBASE, and The Cochrane Library were searched from 2006 to April 2016. English language studies that planned or evaluated guideline implementation targeted to physicians based on explicitly named theory were eligible. Screening and data extraction were done in duplicate. Study characteristics and details about theory use were analyzed.

**Results:**

A total of 1244 published reports were identified, 891 were unique, and 716 were excluded based on title and abstract. Among 175 full-text articles, 89 planned or evaluated guideline implementation targeted to physicians; 42 (47.2%) were based on theory and included. The number of studies using theory increased yearly and represented a wide array of countries, guideline topics and types of physicians. The Theory of Planned Behavior (38.1%) and the Theoretical Domains Framework (23.8%) were used most frequently. Many studies rationalized choice of theory (83.3%), most often by stating that the theory described implementation or its determinants, but most failed to explicitly link barriers with theoretical constructs. The majority of studies used theory to inform surveys or interviews that identified barriers of guideline use as a preliminary step in implementation planning (76.2%). All studies that evaluated interventions reported positive impact on reported physician or patient outcomes.

**Conclusions:**

While the use of theory to design or evaluate interventions appears to be increasing over time, this review found that one half of guideline implementation studies were based on theory and many of those provided scant details about how theory was used. This limits interpretation and replication of those interventions, and seems to result in multifaceted interventions, which may not be feasible outside of scientific investigation. Further research is needed to better understand how to employ theory in guideline implementation planning or evaluation.

**Electronic supplementary material:**

The online version of this article (doi:10.1186/s13012-017-0557-0) contains supplementary material, which is available to authorized users.

## Background

Clinical practice guidelines are widely developed to inform and augment health care policy, planning, delivery, evaluation, and quality improvement. Guidelines offer many potential benefits to patients, health care professionals, and health systems by supporting decision-making and enhancing the efficiency and quality of health services, while reducing practice variation [1]. However, numerous population-based studies have shown that guideline implementation is complex and challenging [2, 3] due to the influence of numerous, multilevel (patient, provider, team, organization, system), often competing factors [4]. Considerable research has been undertaken over the last three decades to identify effective single or multifaceted interventions for implementing guidelines [5–7]. While many of those interventions are promising, their impact on health care delivery and outcomes has been modest and inconsistent [8, 9]. Given the important role of guidelines in translating scientific evidence to practice, further research is needed to generate knowledge on how to optimize guideline implementation and use.

To address this need, the field of implementation science has pursued research in two predominant themes. One theme focused on elaborating and refining the guideline implementation planning process so that the most promising interventions for a given context are employed [10]. Another theme focused on identifying the interventions and active components of interventions that are associated with effective guideline implementation so that, when employed, they result in beneficial impact. In 2005, a Cochrane systematic review by Shaw et al. found that guideline implementation interventions selected and tailored according to the advance identification of potential barriers of guideline use were more likely to improve professional practice compared with either no intervention or the dissemination of guidelines [11], and this was supported by subsequent research [12]. As a result, implementation scientists advocated for choosing and adapting interventions based on mapping pre-identified determinants, including barriers and facilitators of guideline use, with theoretical constructs [12]. Theories (or models or frameworks) suggest how determinants influence the association between processes and outcomes and provide insight on interventions (approaches, strategies) that overcome determinants and/or support processes associated with desirable outcomes [13].

However, guideline developers, implementers, researchers, or others may not be using theory when planning or undertaking guideline implementation. A systematic review published in 2010 by Davies et al., based on controlled trials, before-after studies and interrupted time series that evaluated any guideline dissemination or implementation strategy targeting physicians and reported objective measures of provider behavior and/or patient outcomes, found that only 6.0% (14/235) of studies of guideline implementation planning or evaluation were explicitly based on theory [14]. Those studies were published from 1976 to 1998, well before knowledge was published of the need to tailor interventions to identified determinants of guideline use [11]. Hence, guideline developers, implementers, researchers or others may not be aware of, or be employing the most relevant theories from among the multitude that are available [15], or may not understand how to use theory when interpreting identified determinants, or choosing or tailoring interventions [16]. Theory may be more commonly used in recently published research, although this is unknown. Furthermore, recent rigorous studies that evaluated tailored, theory-based interventions in various health care contexts have failed to consistently demonstrate a beneficial impact on physician use of guidelines or patient outcomes [17, 18]. Theory use may be sub-optimal, but without further analysis of such studies, this too is unknown.

Considerable resources continue to be invested in the development of guidelines that are not achieving the maximum benefit of which they are capable, potentially due to limitations in the way they are implemented. Further research is needed to understand if theory is being used to plan, undertake, and evaluate guideline implementation. In particular, greater insight is needed on how theory was employed in guideline implementation research. This may reveal the need for interventions to promote awareness, knowledge, and skill in the use of theories for guideline implementation, or for further research on how to use theory in guideline implementation. The purpose of this scoping review was to summarize current research in the field of guideline implementation to describe if and how theory has been used to plan or evaluate the implementation and use of guidelines among physicians, who are frequently the target users of guidelines.

## Methods

### Approach

This review sought to identify theories that were used to plan or evaluate guideline implementation targeted to physicians, reveal rationale for choice of theory as is required by the Workgroup for Intervention Development and Evaluation Research (WIDER) reporting standards for studies that evaluate behavioral interventions [19] and describe how theory was used. Therefore, rather than a traditional systematic review that seeks to describe outcomes, a five-step scoping review was chosen: scoping, searching, screening, data extraction, and data analysis [20]. This approach was employed to acquire an understanding of the extent, range, and nature of research on this topic, and to describe if and how theory has been used to plan or evaluate guideline implementation. The Preferred Reporting Items for Systematic Reviews and Meta-Analyses (PRISMA) criteria guided the conduct and reporting of this review [21]. Data were publicly available so institutional review board approval was not necessary. A protocol for this review was not registered.

### Scoping

As per scoping methods standards, this step involved becoming familiar with the literature on this topic and generated eligibility criteria through consultation with the members of the international research team who are experienced guideline developers and implementers. A preliminary search was conducted in MEDLINE using Medical Subject Headings (MeSH) including, but not limited to, diffusion of innovation or information dissemination and practice guidelines as topic or guideline adherence and a range of available terms that referred to theory (for example, models, theoretical). LL, ARG, SB, and RV screened titles and abstracts of the preliminary search results, which were used to plan a more comprehensive search strategy and to generate final eligibility criteria based on the PICO (population, intervention, comparisons, outcomes) framework. All members of the research team reviewed eligibility criteria and provided feedback, which was used to refine the eligibility criteria. The research team was comprised of guideline developers, implementation scientists, and systematic review methodologists.

*Populations* referred to practicing physicians of any type based in health care settings (e.g., hospitals, ambulatory clinics, community-based physician offices) who were target users of specifically named and referenced guidelines upon which implementation efforts were focused, either for guidelines newly developed, or to improve compliance with an existing guideline. Studies were included if the target users were of various professions provided that the majority were physicians. Guidelines referred to clinical practice guidelines, defined as statements that include recommendations intended to optimize patient care based on systematic review of evidence, for any form of test, procedure, or treatment for any condition or disease, that were specifically named and cited in eligible primary studies [22].

*Interventions* referred to guideline implementation processes or interventions in which theory was explicitly named, referenced, and used. Theory was defined as a set of analytical principles or statements including defined variables, a domain to which the theory applies, and a set of relationships between the variables and specific predictions [23]. Theory-informed frameworks or models were also considered. Interventions also referred to the strategies or processes that were chosen, tailored, implemented, or evaluated to promote or improve guideline use. Similar to the 2010 systematic review of the use of theory in guideline implementation [14], eligible studies used theory to (1) identify potential determinants of guideline use among target users with questionnaires or qualitative methods or analyze the findings of such studies; (2) choose or tailor interventions that would address identified determinants, either among the research team or through structured consultation with experts or stakeholders (Delphi, modified Delphi, nominal group, etc.); or (3) evaluate the impact of interventions, including studies that examined implementation (fidelity), guideline use (practice/behavior), or impact (patient, provider, organizational, or system-level outcomes).

*Comparisons* varied depending on the type of study. For example, in studies of type 1, identified determinants may have been similar or varied by specialty or practice setting. In type 2 studies, the choice of intervention or tailoring strategy may have been dependent on characteristics of the involved participants. Type 3 studies may have evaluated physicians with and without exposure to theory-based interventions, or before or after exposure to theory-based interventions, or receiving different types of theory-based interventions, or compared any type of theory-based intervention with any type of non-theory based intervention or control condition, where interventions may have been single or multifaceted.

*Outcomes* were those reported in eligible studies and were also relevant to study type: (1) perceived or experienced determinants of guideline use including recommended facilitators or interventions to promote guideline use; (2) recommended interventions or strategies to tailor interventions; and (3) any reported impact of guideline implementation.

Eligible study designs included English language qualitative (interviews, focus groups, qualitative case studies), quantitative (questionnaires, randomized controlled trials, time series, before-after studies, prospective or retrospective cohort studies, case control studies), or mixed methods studies (studies that integrated quantitative and qualitative data). Systematic reviews were not eligible but were used to identify additional eligible primary studies. Studies were not eligible if they:

- involved trainee physicians, health care managers or policy-makers, or consumers (patients, families, caregivers, public) as target guideline users
- were based on infection control, quality improvement, patient safety, client-centeredness, or organizational “best practices” but did not explicitly name and reference a guideline
- were based on grounded theory technique (a qualitative approach) and did not analyze the findings using an explicitly named and referenced theory, model, or framework
- searched for, described, developed, or synthesized theory without applying it to plan or evaluate guideline implementation
- referenced a theory, model, or framework but made no further mention of it
- were based in a school or sports setting
- concluded that an intervention was needed to address lack of compliance with a guideline
- or consisted of policy directives or strategies, consensus statements, guidelines, conference abstracts or proceedings, protocols, letters, editorials, or commentaries.

### Searching

A final comprehensive search strategy compliant with the Peer Review of Electronic Search Strategies statement [24] was developed in consultation with a medical librarian (Additional file 1). MEDLINE, EMBASE, and The Cochrane Library were searched on April 26, 2016 for articles published from January 2006 to that date. The year 2006 was chosen because evidence supporting the need to choose and tailor interventions for implementing guidelines based on identified determinants [11] and relevant theories [12] was published by Shaw et al. in 2005. As noted, the references of systematic reviews were scanned to identify additional eligible articles.

### Screening

For the ultimate search results, the screening of titles and abstracts according to specified eligibility criteria was independently piloted by LL, ARG, SB, and RV. LL and RV then independently screened titles and abstracts of all non-duplicate items. All items selected by at least one reviewer were retrieved for full-text screening. Full-text items were screened just prior to data extraction by LL in consultation with ARG. If more than one publication described a single study and reported different data, they were all included but counted as a single study.

### Data extraction

A data extraction form was developed with input from the research team to collect information on study characteristics (author, publication year, country, design), condition/disease, physician specialty, guideline topic, aim (implement new guideline or improve compliance with existing guideline), theory (or model or framework) used, rationale for choice of theory as specified in individual studies (describes implementation or its determinants, used by others, validated, integrates many theories and/or constructs), and how theory was used (identify determinants, select/tailor interventions, evaluate impact of intervention) [14]. Qualitative details were extracted about how theory was used if provided. We did not access related studies previously published by the same authors that may have reported these details because reporting standards specify that theory underlying the development or evaluation of interventions should be specified when reporting research [19]. Details extracted about the intervention were based on the Workgroup for Intervention Development and Evaluation Research (WIDER) reporting checklist and included content (information/knowledge conveyed), format (mode of delivery), timing (duration and/or frequency), participants (number, type, setting), and personnel (who delivered the intervention) [19]. Interventions were classified as single or multifaceted. Outcomes varied by study type: (1) identified determinants, (2) intervention chosen, and strategies or process for tailoring, and (3) impact of the intervention. LL and ARG independently pilot-tested data extraction on two articles and compared findings by discussion to refine the data extraction form. This was repeated two more times. The refined data extraction form was independently pilot-tested by LL, SB, and RV on five articles. LL extracted data from all articles, which was independently checked in by ARG, SB, and RV so that all were reviewed in duplicate.

### Data analysis

Summary statistics (i.e., frequency, proportion) were used to describe the number of studies by year published, country, research design, guideline topic, and type of target user. Each unique theory (or model/framework) was listed, and summary statistics were used to report the frequency of use, rationale for use, and how theories were used. Theories were classified as classic (originating from fields external to implementation science such as psychology, sociology, organizational management) or implementation theories (developed de novo by implementation researchers or by adapting existing classic theories) [23]. Details about interventions that were evaluated and associated outcomes were charted (collated, synthesized, and interpreted) and summarized in a narrative format [25]. The quality of individual studies was not assessed because that is not customary for a scoping review. All co-authors reviewed the summary of findings, and their feedback was incorporated in the final version.

## Results

### Search results

**Fig. 1:**
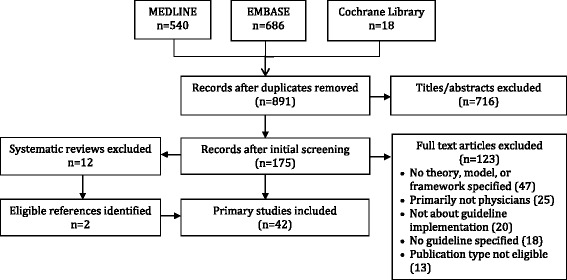
PRISMA diagram of study selection and inclusion

A total of 1244 studies were identified by searches, of which 891 were unique items, and 716 were excluded based on screening of titles and abstracts. Among 175 full-text articles that were screened, 123 were excluded because a theory, model, or framework was not named or referenced (47), target users were not predominantly physicians (25), the focus was not guideline implementation (20), a guideline was not named or referenced (18), or because the publication type was not eligible (13). An additional 12 studies were excluded because they were systematic reviews, from which 2 eligible studies were identified among references to primary studies. A total of 42 studies were included in the review (Fig. 1). While 42 studies were included in this review because they did employ a theory, of 123 full-text studies that were excluded, 47 were excluded because they were otherwise eligible but did not employ a theory. Therefore, of 89 studies that planned or evaluated guideline implementation targeted to physicians, 47.2% were based on theory. Data extracted from included studies are available in Additional file 2 [26–67].

### Study characteristics

**Fig. 2:**
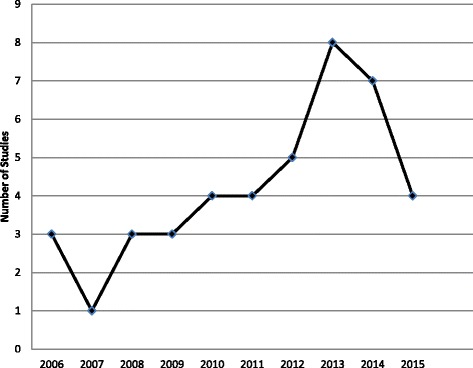
Number of included studies published per year

Studies were published between 2006 and 2015. The number of studies published per year increased almost annually from 2006 to 2013 and then declined in 2014 and again in 2015 (Fig. 2). Studies were conducted in the United Kingdom (10, 23.8%), Australia (9, 21.4%), the United States (7, 16.7%), the Netherlands (6, 14.3%), Canada (3, 7.1%), Iran (3, 7.1%), Argentina (1, 2.4%), Belgium (1, 2.4%), Germany (1, 2.4%), and Saudi Arabia (1, 2.4%). Most studies used a single cohort design (34, 81.0%). Of these, 18 employed questionnaires, 10 used interviews, 3 were based on focus groups, 2 were before-after evaluations of an intervention, and 1 described the intervention that was employed. The remainder (7, 16.7%) were randomized controlled trials (RCTs) and one mixed methods study (2.4%).

### Guidelines

Guidelines targeted in included studies pertained to a wide variety of health care topics. The diseases or conditions most frequently addressed included asthma (5, 11.9%), mental health (4, 9.5%), antibiotic prescription (3, 7.1%), coronary heart disease (3, 7.1%), diabetes (3, 7.1%), and osteoarthritis (3, 7.1%). The remaining studies targeted various health care issues. The majority of studies were planning or evaluating the implementation of an existing guideline to improve compliance (38, 90.5%) rather than planning or evaluating the implementation of a newly developed guideline.

### Target users

The users most frequently targeted were general practitioners (17, 40.5%) followed by multidisciplinary groups of physicians (14, 33.3%). The remaining studies targeted various physician specialties (11, 26.2%).

### Theories used

Table 1 summarizes the number and type of theories used in included studies. The Theory of Planned Behavior (TPB), a classic theory (16, 38.1%), and the Theoretical Domains Framework (TDF), an implementation theory (10, 23.8%), were the most frequently used theories. Normalization Process Theory, an implementation theory, was used in two studies [29, 32]. Other theories employed were all classic in origin and included Diffusion of Innovation Theory [49, 61, 65], Social Cognitive Theory [58, 60], Adult Learning Theory [33], Social Marketing Theory [39], Social Learning Theory [55], Self-Perception Theory [60], and Fuzzy-Trace Theory [67]. Several studies used models (6, 14.3%) or frameworks (4, 9.5%). Models or frameworks used more than once included the Cabana Framework of Barriers to Physician Guideline Adherence [44, 52, 56] and the Attitude Social Norm Self Efficacy model [43, 53]. Some studies used a combination of two or more theories, models, or frameworks [40, 55, 60], two of which used one or both of the TPB or TDF [40, 55].

**Table 1:** Number, type, and use of theories in included studies

Theory (or models/frameworks)	Employed in included studies, *n* (% of 42)	How used *n* (%)		
		identify barriers (% of 32 studies)	Select and/or tailor intervention (% of 2 studies)	Evaluate intervention impact (% of 8 studies)
Theory of Planned Behavior	16 (38.1)	14 (43.8)	–	2 (25.0)
Theoretical Domains Framework	10 (23.8)	8 (25.0)	1 (50.0)	1 (12.5)
Diffusion of Innovation Theory	3 (7.1)	2 (6.3)		1 (12.5)
Cabana Framework of Barriers to Physician Guideline Adherence	3 (7.1)	3 (9.4)	–	–
Social Cognitive Theory	2 (4.8)	–	–	2 (25.0)
Normalization Process Theory	2 (4.8)	1 (3.1)	–	1 (12.5)
Attitude Social Norm Self Efficacy Model	2 (4.8)	1 (3.1)	1 (50.0)	–
Adult Learning Theory	1 (2.4)	–	1 (50.0)	–
Social Marketing Theory	1 (2.4)	–	–	1 (12.5)
Social Learning Theory	1 (2.4)	–	–	1 (12.5)
Self-Perception Theory	1 (2.4)	–	–	1 (12.5)
Fuzzy-Trace Theory	1 (2.4)	1 (3.1)	–	–
Dual Process Model of Behavior	1 (2.4)	1 (3.1)	–	–
Knowledge Attitude Behavior Framework	1 (2.4)	1 (3.1)	–	–
Elaboration Likelihood Model	1 (2.4)	–	–	1 (12.5)
Social Influence Model of Behavior Change	1 (2.4)	–	–	1 (12.5)

### Rationale for theories selected

Several studies (7, 16.7%) referenced a theory, model, or framework but provided no rationale for its selection. Most studies (33, 83.3%) provided an explanation for the choice of theory, model, or framework employed. Rationales were grouped into four categories that emerged from the data. The theory (or model/framework) describes implementation, or its determinants was the rationale referred to by most (31/35, 88.6%) studies, for example, “The constructs of the TPB can be used as a framework to explore theoretically derived determinants of behavior” [38]. Other studies that provided this rationale were less clear, for example, theories “address both the how and why of change” [55]. Some studies explicitly stated that the theory (or model/framework) was previously used by others (17/35, 48.6%) or had been validated (14/35, 40.0%), for example, “The TDF has been validated and used in multiple health care settings to assist with the systematic identification of barriers and enablers to implementation” [26]. Other studies noted that the theory was chosen because it integrated many theories or theoretical constructs (5/35, 14.3%), for example, “The TDF aims to synthesize a multitude of coherent behavior change theories into a single framework that allows assessment and explanation of behavioral problems and associated barriers and enablers, and inform the design of appropriately targeted interventions” [36]. Overall, while many studies provided more than one rationale (24/35, 68.6%), in most cases the reasons were not explicitly linked with identified barriers or facilitators of guideline use so that the reader could clearly understand how theoretical constructs predicted or explained context-specific conditions that challenged guideline implementation.

### How theories were used

Table 1 summarizes how theories were used in included studies. The majority of studies were type 1 (32, 76.2%). These studies used theory to identify determinants of guideline use as a preliminary step in guideline implementation planning. Of these, 15 studies used theory to inform survey questions, while 4 studies used theory to analyze survey findings. Another 6 type 1 studies used theory to inform questions that were used during qualitative interviews or focus groups; 6 studies used theory to analyze interview or focus group findings; and 1 study used theory to both inform interview questions and to analyze the findings that emerged from those interviews. Among the 32 type 1 studies, the most frequently used theories (or models/frameworks) were the TPB (14), TDF (7), Cabana Framework of Barriers to Physician Guideline Adherence (3), and the Diffusion of Innovations Theory (2).

Two studies (4.8%) were type 2 [33, 53]. This type of study used theory to select and/or tailor interventions but did not proceed to evaluate the impact of interventions. They both proposed multifaceted interventions. One study employed the Attitude Social Norm Self Efficacy model to analyze the findings of physician interviews about depression guidelines [53]. Identified determinants of guideline use then informed the selection and design of a multifaceted intervention as part of a systematic intervention mapping process. The proposed intervention included educational meetings, educational materials, audit and feedback, and ongoing training. Another study employed the TDF and Adult Learning Theory to design an intervention comprised of 4 workshops of 1 or 2h, delivered from 2 to 3 weeks apart and including didactic, interactive, and role play components plus intervening videos of simulated patient consultations to improve the management of osteoarthritis [33].

Several studies were type 3 (8, 19.0%). These studies evaluated the impact of interventions. One study did not report how theory (Diffusion of Innovation) was used [61]. The remainder employed a range from one to three theories (or models/frameworks). No theory (or model/framework) predominated; they included Normalization Process Theory [32], TDF [40], Social Marketing Theory [39], TPB [40, 55], Social Learning Theory [55], Social Cognitive Theory [58, 60], Elaboration Likelihood Model [60], Self-Perception Theory [60], Diffusion of Innovation Theory [61], and Social Influence Theory [64]. In type 3 studies, theories were used in different ways including to design interventions [39, 55, 58, 60, 64], to select interventions [40, 58, 60], to identify determinants [40, 58], and to evaluate the findings of interviews that probed for the acceptability and feasibility of the intervention [32, 55]. One study that used theory to identify determinants and select and design the intervention did so through use of a formal intervention mapping process [58].

### Interventions evaluated

Additional file 3 provides details on the eight type 3 studies that evaluated the impact of interventions, which were selected, designed, and/or evaluated based on theories (or models/frameworks) [32, 39, 40, 55, 58, 60, 61, 64]. Two of those studies, both randomized controlled trials, evaluated single interventions [40, 60]. One was based on a mixed didactic and interactive educational workshop on the management of low back pain and employed the TDF and TPB [40]. It found that intervention group physicians had significantly greater intention of complying with the guideline for X-ray referral, adhered with guideline recommendations for X-ray referral, and were more likely to give advice to stay active; there were no differences between intervention and control physicians with respect to referral for imaging. Another study addressed smoking cessation counseling and employed Social Cognitive Theory, Self-Perception Theory, and Elaboration Likelihood Model [60]. It included three intervention arms, each based on a different single intervention including educational information, a quiz with educational information provided as answers, and feedback of self-reported performance data. Compared with control, only those in the educational information group reported significantly higher intention and more frequently recommending smoking cessation.

Six studies, including two qualitative studies embedded in RCTs [32, 55], two single cohort before-after studies [39, 58], and two RCTs [61, 64] evaluated multifaceted interventions. Two qualitative studies assessed the implementation fidelity of interventions. In one, which employed Normalization Process Theory, participating GPs who were interviewed identified several benefits of the intervention, comprised of a series of four workshops including didactic, interactive, and role play components [32]. They said that they better understood how to apply osteoarthritis guidelines and were comfortable with the clinical intervention that included a guidebook for patients and referral to nurse-led clinics. In the other qualitative study, which employed TPB and Social Learning Theory, based on a trial of a seven-part intervention that included online modules and an in-person seminar, interviewed GPs reported improved self-efficacy and changes in consultation style and antibiotic prescribing [55]. Participants appreciated receiving current scientific evidence and their own performance data.

Two single cohort studies both achieved positive impact. Educational outreach plus educational information significantly increased physician knowledge, intent to use venous thromboembolism (VTE) prophylaxis, and number of patients receiving VTE prophylaxis in a study that employed Social Marketing Theory [39]. Educational outreach plus educational information, together with appeals to professional associations, formulary systems, and mass media significantly increased intent to prescribe diuretics for hypertension management in a study that employed Social Cognitive Theory [58].

Two RCTs also reported positive impact. One RCT, which employed Diffusion of Innovation Theory and engaged birth attendants as opinion leaders who disseminated guidelines, provided training, and generated and shared monthly performance data [61]. Readiness to change and prophylactic use of oxytocin increased significantly, and the rate of episiotomy decreased significantly in intervention hospitals compared with control hospitals. In a second RCT of educational outreach visits, which employed Social Influence Model of Behavior Change, computer reminders, telephone follow-up, and a financial incentive, screening, the number of skin tests, detection of active cases of tuberculosis, referral of patients with tuberculosis, and use of vaccine increased significantly in intervention practices compared with control practices [64].

## Discussion

This study was conducted to understand if and how theory (or models/frameworks) was used to plan or evaluate guideline implementation, as has been advocated [11, 12]. Of 89 studies that planned or evaluated guideline implementation targeted to physicians, nearly half (42, 47.2%) were based on theory and included in this scoping review. This compares favorably with the Davies et al. 2010 review that reported explicit theory use in 6.0% of guideline implementation studies published from 1976 to 1998 [14]. There does appear to be an upward trend because, in our review, the number of published studies meeting our inclusion criteria increased almost yearly and represented a wide array of countries, guideline topics, and types of target physicians. However, many studies were excluded because they did not use theory, several studies cited theory but made no further mention of it, and not all studies of each type (identify determinants, select/tailor interventions, evaluate interventions) employed theory or explicitly linked pre-identified determinants of guideline use with theory. Overall, theory was not used consistently and transparently in guideline implementation.

A few issues may limit the interpretation and use of these findings. Although we searched the most relevant databases of medical literature pertaining to physicians with a search that complied with standards [24] and employed rigorous searching and screening processes, we may not have identified all relevant studies. We focused on physicians, who are key target users of guidelines, as did the prior Davies et al. study of theory use in guideline implementation [14] and therefore potentially excluded relevant studies in which non-physicians were target users. Whether these results transfer to other health professionals requires investigation. Publication bias, or the tendency for journals to publish trials with positive results or surveys with high response rates, may have influenced the number and type of studies that were retrieved and the largely positive impact of eligible studies that evaluated interventions. We did not thoroughly discuss the impact of interventions because the purpose of this scoping review was not to assess the effectiveness of interventions, but to describe how theory was used when planning or evaluating guideline implementation.

Several notable findings emerged from this review. While use of theory to plan or evaluate guideline implementation increased subsequently to the review published in 2010 by Davies et al. [14], many studies were not based on theory, or cited theory but made no further mention of how it was used. A previous systematic review of 32 studies published from 2004 to 2013 that evaluated the implementation of guidelines for arthritis, diabetes, colorectal cancer, and heart failure also found that few studies rationalized intervention choice by referring to models, frameworks, or theories (6/32, 18.8%) [68]. Another systematic review of 57 studies published from 1990 to 2000 evaluated the adoption of health care innovations, including guidelines and reported that none of the studies employed theory [69]. Other researchers have also found limited use of theory to design or evaluate public health interventions [70], to inform the implementation of guidelines targeted to community pharmacists [71] or to plan interventions targeted to the allied health professions [72]. Theory-driven implementation is considered a required standard, yet many intervention developers are not using theory. Further research is needed to establish whether those who implement guidelines are familiar with theories and how to apply them. Such research could also examine if education or discipline of the implementers are associated with use of theory, and whether or not including health services researchers are familiar with theory to guideline teams improves theory-informed implementation.

Another key finding is that, while most studies justified the selection of theories, the rationales provided were lacking in specificity and failed to explicitly link identified context-specific determinants with particular theoretical constructs. This too has been identified by others in various health care contexts. For example, in a systematic review of 62 studies that used theory to design or evaluate public health interventions, descriptions of theory development and use in intervention design and evaluation lacked detail [70]. Another systematic review of 32 studies that evaluated interventions targeted to allied health professionals reported that most studies did not describe how the interventions were developed or their underlying mechanism of action [72]. The Workgroup for Intervention Development and Evaluation Research (WIDER) criteria for reporting of knowledge translation interventions [19] and the Template for Intervention Description and Replication (TIDieR) checklist for better reporting of interventions [73] both recommend the inclusion of the rationale, theory, or change process that underpins an intervention as this can help others to know which elements are essential, rather than optional or incidental [73]. Intervention mapping, used by only two studies included in this review [53, 58], is an increasingly used process for engaging stakeholders in choosing and designing theory-based interventions [74, 75]. It offers a systematic and explicit process for mapping identified determinants to program objectives, and selecting evidence- and theory-informed interventions likely to achieve those objectives, which may help intervention developers to better report how theory was used. Further research is needed to understand whether guideline implementers are aware of reporting criteria such as WIDER and TIDieR, or processes such as intervention mapping. However, while all of these resources specify that the use of theory and its rationale are needed and should be explicit, they do not actually provide guidance on how to choose and apply theory. Thus, the development of more detailed reporting guidance specific to the use of theory is needed.

This review found that all eight studies that evaluated the impact of interventions achieved positive impact on the outcomes reported. Yet, many others have not [15, 16]. A recent process evaluation of five failed trials that evaluated theory-based tailored interventions for guideline implementation found that only some of the determinants targeted by the intervention were relevant to participants who identified many new barriers that were not addressed by the intervention [76]. It may be that pre-determined barriers do not cover all factors that potentially affect implementation outcomes; that other strategies for identifying barriers and facilitators are necessary; that the removal of one barrier may create another one; and that the complex interplay among various barriers and facilitators cannot always be predicted despite best efforts for doing so [77]. These issues raise several implications—is the use of a standardized theory-driven approach to implementation planning problematic because it cannot accommodate the reality of the fluidity of barriers and facilitators? Or are theories from a variety of disciplines needed that differ from those that have been historically used to account for the complexity of implementation? Or is there a limit as to what we can expect from theory? Further research is needed to more fully understand why theory-informed interventions fail to consistently achieve desired outcomes and to address these related questions.

Based on the studies in our review that proposed or evaluated an intervention, it appears that the use of theory commonly gives rise to multifaceted interventions. In other research, a meta-review of 25 systematic reviews that compared direct and indirect effect size and dose-response of single and multifaceted strategies showed no benefit of multifaceted over single strategies [78]. Similar findings emerged from a systematic review of studies that evaluated the implementation of neck and/or back pain guidelines [79]. These findings contrast with the prevalent use of theory-informed multifaceted guideline implementation interventions by studies included in this review. In comparison with single interventions, multifaceted interventions may be more expensive, may place a higher burden on those delivering and receiving the intervention, and may not be easily replicable outside of the context of scientific investigation. Therefore, further research is needed to better understand how to employ theory when designing or evaluating guideline implementation so that interventions are feasible and can be more readily scaled up if found to be effective, which is the ultimate end-goal of implementation science.

TPB and TDF emerged as the most commonly used theories giving rise to multifaceted interventions. These were chosen because they had been used by others and because they addressed a broad range of determinants, rather than explicitly matching pre-identified determinants to specific theory chosen from among the many that are available. Therefore, guideline implementers may benefit from information about theories and how to use them. To supply this information, research is needed to more firmly establish which theories and how many theories (or models/frameworks) result in effective interventions. In addition, educational interventions may be needed to enhance guideline implementer awareness of existing compilations of theories from various disciplines [15, 80–83].

## Conclusions

This scoping review of studies published from 2006 to 2015 that planned or evaluated guideline implementation targeted to physicians found that nearly half were based on theory (or models/frameworks). This compared favorably with previous studies. The majority of studies employed the TPB or the TDF and used theory to inform survey or interview questions that identified determinants of guideline use. Positive outcomes were achieved by the few studies that evaluated interventions, the majority of which were multifaceted. However, most studies provided few details about why the theory was chosen or how it was used and, in particular, did not explicitly link pre-identified determinants of guideline use to specific theoretical constructs. Further research is needed to assess awareness and knowledge of theory among guideline implementers, to develop a more detailed guidance on the use and reporting of theory-informed implementation, to establish the number of types of theories that result in improved guideline use, to understand why theory-informed interventions fail to consistently achieve desired outcomes, and to generate insight on how to design theory-based interventions that are feasible to broadly apply outside of the research context.

## Additional files

Additional file 1: MEDLINE search strategy. (DOCX 31 kb)
Additional file 2: Characteristics of included studies. (DOC 127 kb)
Additional file 3: Description of interventions evaluated in included studies. (DOC 58 kb)
