# Supplementary material for: Use of theory to plan or evaluate guideline implementation among physicians: a scoping review
Source: Implement Sci. 2017 Feb 27;12:26. doi: 10.1186/s13012-017-0557-0 (PMC5327520; doi:10.1186/s13012-017-0557-0)
Supplement: Additional file 1: — MEDLINE search strategy. (DOCX 31 kb) [file 13012_2017_557_MOESM1_ESM.docx]

Records after duplicates removed

(n=891)

Titles/abstracts excluded

(n=716)

Full text articles excluded

(n=123)

- No theory, model, or framework specified (47)
- Primarily not physicians (25)
- Not about guideline implementation (20)
- No guideline specified (18)
- Publication type not eligible (13)

Records after initial screening

(n=175)

Primary studies included

(n=42)

MEDLINE n=540

EMBASE n=686

Cochrane Library n=18

Systematic reviews excluded

n=12

Eligible references identified

n=2
