# Supplementary material for: Use of theory to plan or evaluate guideline implementation among physicians: a scoping review
Source: Implement Sci. 2017 Feb 27;12:26. doi: 10.1186/s13012-017-0557-0 (PMC5327520; doi:10.1186/s13012-017-0557-0)
Supplement: Additional file 2: — Characteristics of included studies. (DOC 127 kb) [file 13012_2017_557_MOESM2_ESM.doc]

Additional File 2. Characteristics of included studies (n =42)

| Study | Research design | Health care issue | Guideline topic (new or existing) | Physician specialty | Theory (model or framework) | Type of theory | Rationale for theory (rationale category) | Type of study and Use of theory | Type of intervention planned or evaluated |
| --- | --- | --- | --- | --- | --- | --- | --- | --- | --- |
| Barlow 2015 UK  [26] | Mixed methods (questionnaire and interviews) | Osteoarthritis | Arthroscopy in knee osteoarthritis (existing) | Surgeons (orthopaedic consultants, registrars, associate specialists, and staff grades) | TDF | Implementation | The TDF has been validated and used in multiple health care settings to assist with the systematic identification of barriers and enablers to implementation (validated, used by others) | Type 1: The questionnaire data were mapped to the TDF (informed interview questions) | N/A |
| Phipps 2015 UK  [27] | Single cohort (questionnaire) | Anaesthetic procedural violations | Anaesthetic practice (existing) | Anaesthetists | TPB | Classic | One paradigm that has been used to capture such influences on rule-related behaviour, and to suggest interventions for dealing with violations, is the theory of planned behaviour (used by others, describes implementation or its determinants) | Type 1: The questionnaire was based on the TPB (inform survey questions) | N/A |
| van Peet 2015 United States  [28] | Single cohort (focus group) | Cardiovascular disease | Secondary cardiovascular prevention in older age (existing) | General practitioners | TDF | Implementation | NR | Type 1: The TDF was used for deductive coding of group discussions (analyze focus group findings) | N/A |
| Vest 2015 United States  [29] | Single cohort (interview) | Chronic kidney disease | Identification and management of chronic kidney disease (existing) | General practitioners, nurse practitioner, physician assistant | NPT | Implementation | The NPT has proven useful in understanding the work involved in implementing and integrating new practices into health care settings. NPT provides a framework for examining implementation processes by dividing the "work" of integrating new practices into 4 domains (used by others, describes implementation or its determinants) | Type 1: Themes from interviews were coded according to the NPT (analyze interview findings) | N/A |
| Chaves 2014 Australia  [30] | Single cohort (questionnaire) | Antimicrobial use | Antibiotic prescribing guideline (existing) | Interns, residents, registrars, consultants | TDF | Implementation | The TDF has been used to identify barriers and enablers and inform behavior change strategies in several different healthcare disciplines (used by others, describes implementation or its determinants) | Type 1: Findings were mapped to TDF domains (analyze survey findings) | N/A |

| Study | Research design | Health care issue | Guideline topic (new or existing) | Physician specialty | Theory (model or framework) | | Type of theory | | Rationale for theory (rationale category) | | Type of study and Use of theory | | Type of intervention planned or evaluated | |
| --- | --- | --- | --- | --- | --- | --- | --- | --- | --- | --- | --- | --- | --- | --- |
| Murphy 2014 Australia  [31] | Single cohort (interview) | Dementia | Detection, diagnosis, and management of dementia (existing) | General practitioners managing people with suspected cognitive impairment or dementia | TDF | | Implementation | | The TDF is an integrated theoretical framework that has been validated and used in a number of empirical studies (integrates many theories, validated, used by others) | | Type 1: Semi-structured interviews based on the TDF (inform interview questions) | | N/A | |
| Ong  2014  UK  [32] | Qualitative focus group study embedded in a cluster-randomized controlled trial | Osteoarthritis | Treatment and self-management of osteoarthritis (existing) | General practitioners | NPT | | Implementation | | NPT examines the processes of change in context, considers the multi-layered reality and overlapping processes affecting behaviour change and attempts to capture the extreme ends of the process from sense-making to routinisation. NPT is particularly effective at unpicking if and how people make sense of complex interventions in primary care (describes implementation or its determinants, used by others) | | Type 3: The element of coherence from the NPT framework informed data collection and analysis of physician interviews that addressed feasibility of the intervention including barriers and facilitators | | Clinical intervention: GP consult, provision of OA guidebook, referral to a nurse-led OA clinic, patient chart template to code OA consult  Behavioural intervention: 4 educational workshops and computer reminder | |
| Porcheret 2014 UK  [33] | Single cohort | Osteoarthritis | Treatment of osteoarthritis (existing) | General practitioners, practice nurses | TDF, adult learning theory | | Implementation + Classic | | The TDF was selected to facilitate understanding of the determinants that could facilitate or impede the intended change. It encompasses many constructs and theories, has been used for an extensive range of conditions and clinical situations, and has been recently validated and refined (integrates many theories, used by others, validated). Adult learning theory has a well-established role in continuing professional development (used by others) | | Type 2: TDF was used to examine barriers of guideline use that were discussed at three advisory group meetings, and adult learning theory was used to design the delivery/training for the intervention | | Clinical intervention:  an OA Guidebook to provide patient-centred and evidence based  information, an enhanced OA consultation  by GPs and practice nurses, and access to a practice based  nurse-led OA clinic to support self-management  Behavioural intervention: four small group sessions two weeks apart at GP offices of mixed didactic and interactive discussion facilitated by opinion leaders and including communication skills training | |
| Study | Research design | Health care issue | Guideline topic (new or existing) | Physician specialty | | Theory (model or framework) | | Type of theory | | Rationale for theory (rationale category) | | Type of study and Use of theory | | Type of intervention planned or evaluated |
| Presseau 2014 UK  [34] | Single cohort (questionnaire) | Type 2 diabetes | Management of patients with type 2 diabetes (existing) | Primary care doctors and nurses in general practices | | (Dual process model of behavior) | | N/A | | Dual process models propose that behavior results from the interplay of a reflective and an impulsive process. Four decades of research in social and health psychology have demonstrated the importance of the reflective process in determining behavior (validated, describes implementation or its determinants) | | Type 1: The ability of the dual process model to predict for six guideline-recommended behaviors was tested (informed survey questions) | | N/A |
| Travender 2014 Australia  [35] | Single cohort (interviews) | Mild traumatic brain injury | Management of mild traumatic brain injury (existing) | Medical doctors, registered nurses, nurse practitioners, and ED directors | | TDF | | Implementation | | The TDF consists of 12 domains that can be considered when exploring influencing factors and designing interventions. The TDF has been validated to confirm the optimal domain structure, content, and labels (validated, describes implementation or its determinants, integrates many theories) | | Type 1: The interview guide was informed by the TDF (inform interview questions) | | N/A |
| Wilkinson  2013 Australia  [36] | Single cohort (questionnaire) | Gestational diabetes mellitus | Nutrition practice guidelines (existing) | Obstetric medicine, obstetrician/gynaecologists, midwifery, diabetes educator, nutrition and dietetics | | TDF | | Implementation | | The TDF aims to synthesise a multitude of coherent behavior change theories into a single framework that allows assessment and explanation of behavioral problems and associated barriers and enablers, and inform the design of appropriately targeted interventions (integrates many theories, describes implementation or its determinants) | | Type 1: The TDF guided the categorization of identified barriers (analyze survey findings) | | N/A |
| Andrews 2013 United States  [37] | Single cohort (questionnaire) | Asthma | Care of children with asthma (existing) | General pediatric and pediatric emergency medicine faculty | | (Knowledge-attitude-behavior framework) | | N/A | | As described in the knowledge-attitude-behavior framework, translating evidence-based guidelines into clinical practice requires overcoming barriers (describes implementation or its determinants) | | Type 1: Survey questions were based on the model (inform survey questions) | | N/A |

| Study | Research design | Health care issue | Guideline topic (new or existing) | Physician specialty | Theory (model or framework) | Type of theory | Rationale for theory (rationale category) | Type of study and Use of theory | Type of intervention planned or evaluated |
| --- | --- | --- | --- | --- | --- | --- | --- | --- | --- |
| Cheung 2013 Canada  [38] | Single cohort study (questionnaire) | Adult resuscitations | Advanced trauma and cardiac life support (existing) | Emergency medicine and anesthesia, general internal medicine, general surgery, critical care | TPB | Classic | The constructs of the TPB can be used as a framework to explore theoretically derived determinants of behavior (describes implementation or its determinants) | Type 1: The interview guide was based on the constructs of the TPB (inform interview questions) | N/A |
| Duff 2013 Australia  [39] | Single cohort study (before-after) | Venous thromboembolism (VTE) | VTE prevention (existing) | Multidisciplinary (Doctors who regularly admit medical patients to study site) | Social marketing theory | Classic | An education outreach visit is based on social marketing theory. It relies on the psychological principles of persuasion to influence clinician behavior and promote evidence based practices (describes implementation or its determinants) | Type 3: Theory informed design of intervention delivery: setting the scene, building trust, two-sided communication, delivering key messages, wrapping up, and providing follow-up | Clinical intervention: VTE prophylaxis  Behavioural intervention: one-to-one educational visit from a trained peer facilitator + educational material + follow-up contact |
| French  2013  Australia  [40] | Randomized controlled trial | Acute low back pain | Management of acute musculoskeletal pain (existing) | General practitioners | TDF, TPB | Implementation+Classic | The TDF was used to identify the barriers and enablers to the target behaviors and guide the choice of intervention components (describes implementation or its determinants). NR for TPB | Type 3: TDF was used to analyze the results of qualitative interviews and identify barriers, and to select intervention components associated with those barriers | Clinical intervention: reduce x-ray referral and recommend physical activity  Behavioural intervention: 3 hour educational workshops that included didactic lecture and small group activities |
| Guerrier 2013 Canada  [41] | Randomized controlled trial (control and intervention group questionnaire) | Acute respiratory tract infections | First and second choices for antibiotics to treat ARTIs (existing) | General practitioners and residents | TPB | Classic | Intention can be predicted with high accuracy by measuring the determinants of the TPB. Evidence from meta-analyses and systematic reviews suggests that the TPB has greater predictive performance of behavioral intention and performance than other socio-cognitive theories (validated, describes implementation or its determinants). | Type 1: The questionnaire was based on the TPB to assess intention to use a guideline (inform survey questions) | N/A |

| Study | Research design | Health care issue | Guideline topic (new or existing) | Physician specialty | Theory (model or framework) | Type of theory | Rationale for theory (rationale category) | Type of study and Use of theory | Type of intervention planned or evaluated |
| --- | --- | --- | --- | --- | --- | --- | --- | --- | --- |
| Mazza 2013 Australia  [42] | Single cohort (focus groups) | Preconception | Preconception care (existing) | General practitioners | TDF | Implementation | Implementation researchers have emphasised that the identification of theoretical domains that are causally related to behavior allows for the appropriate targeting of interventions for increasing guideline implementation. Theoretical domains can be used to identify barriers and enablers and to identify aspects of GPs' behavior which can be targeted for intervention (validated, used by others, describes implementation or its determinants) | Type 1: The questions for the focus group were based on the TDF (inform interview questions) | N/A |
| Schellart 2013 Netherlands  [43] | Single cohort (questionnaire) | Depression | Disability assessment of clients with depression (new) | Insurance physicians | (Attitude – social norm – self-efficacy model) | N/A | Researchers have often used the ASE model to investigate the behavioral aspects of the use of guidelines by physicians. The ASE model has been used to explain the behavior of physicians and patients concerning guidelines in an occupational health context (used by others, describes implementation or its determinants) | Type 1: The model was used as a systemic framework for the identification of behavioral markers predicting the intention to use guidelines (inform survey questions) | N/A |
| Teo  2013  Australia  [44] | Single cohort (interviews) | Antibiotic prescription | Appropriate use of antibiotics for various clinical indications (existing) | Members of the antimicrobial stewardship team (policymakers) and prescribers | (Cabana framework of barriers to physician guideline adherence) | N/A | Cabana’s conceptual framework of barriers to physician adherence to practice guidelines provides a structure in which to understand and report barriers to compliance with policy (describes implementation or its determinants) | Type 1: Results from data analysis from interviews were interpreted using the framework (analyze interview findings) | N/A |

| Study | Research design | Health care issue | Guideline topic (new or existing) | Physician specialty | Theory (model or framework) | Type of theory | Rationale for theory (rationale category) | Type of study and Use of theory | Type of intervention planned or evaluated |
| --- | --- | --- | --- | --- | --- | --- | --- | --- | --- |
| Cortoos 2012 Belgium  [45] | Single cohort study (questionnaire) | Suspected infection | Antibiotic use (existing) | All physicians working in the teaching hospital at the time of the study were eligible. Excluded: anesthesiology, dermatology, ophthalmology, obstetrics–gynecology, radiology, pediatrics, psychiatry, physicians working in lab environments, infectious disease specialists | TPB | Classic | The TPB has already been successfully used several times in the evaluation of medical practice. The theory describes the determinants of individual intentions and behaviors (used by others, validated, describes implementation or its determinants) | Type 1: The TPB was used to construct the questionnaire (inform survey questions) | N/A |
| Islam 2012 Canada  [46] | Single cohort (interview) | Red blood cell transfusion | RBC transfusion (existing) | Intensive care unit physicians | TDF | Implementation | The TDF is derived from 33 commonly used behavioral theories. It can be used to identify perceived determinants of behavior to aid selection of specific theories for further testing or to design theory-informed behavior change interventions (integrates many theories, describes implementation or its determinants) | Type 1: Interview responses were coded into TDF domains (analyze interview findings) | N/A |
| Kramer 2012 Germany  [47] | Single cohort (interviews) | Coronary heart disease | Handling of CHD patients in primary care (existing) | GPs and cardiologists | TPB | Classic | NR | Type 1: The interview guideline was aligned with components of the theory of planned behavior (inform survey questions) | N/A |
| Rashidian  2012  Iran  [48] | Single cohort (questionnaire) | Asthma | Management of asthma (existing) | General practitioners | TPB | Classic | Limited studies have demonstrated that the TPB may be able to help in explaining the variation in physicians’ behavior (was used by others, describes implementation or its determinants) | Type 1: The questionnaire included 47 TPB items (inform survey questions) | N/A |

| Study | Research design | Health care issue | Guideline topic (new or existing) | Physician specialty | Theory (model or framework) | Type of theory | Rationale for theory (rationale category) | Type of study and Use of theory | Type of intervention planned or evaluated |
| --- | --- | --- | --- | --- | --- | --- | --- | --- | --- |
| Wahabi 2012 Saudi Arabia  [49] | Single cohort (focus groups) | Childhood bronchial asthma | Management of childhood bronchial asthma (new) | Pediatric Emergency Department staff (consultant physicians, specialists, residents, nurses) | Diffusion of innovation theory | Classic | NR | Type 1: To determine the main themes of the study, the model of the innovation diffusion theory was used (analyze focus group findings) | N/A |
| Presseau 2011 UK  [50] | Single cohort (questionnaire) | Hypertension | Physical activity advice (existing) | General practitioners and nurses | TPB | Classic | The TPB has been tested across a variety of populations, behaviors, and contexts. It has been frequently used to predict health professional behavior. When compared against other social cognition models, the TPB is the most predictive model (used by others, describes implementation or its determinants, validated) | Type 1: The ability of the TPB to predict physical activity counseling behavior was tested (inform survey questions) | N/A |
| Rashidian 2011 Iran  [51] | Single cohort (questionnaire) | Coronary heart disease | Using statins (existing) | General practitioners | TPB | Classic | Several studies have suggested that the TPB has merits in explaining the attitudes and intentions of providers (used by others, describes implementation or its determinants) | Type 1: TPB items were measured on 7-point bipolar or unipolar scales (inform survey questions) | N/A |
| Van Den Boogaard 2011 Netherlands  [52] | Single cohort (interviews) | Subfertile couples | Fertility care (existing) | Gynaecologists and fertility doctors | (Cabana framework of barriers to physician guideline adherence) | N/A | NR | Type 1: The models were used to guide interviews and analyses (inform interview questions and analyze interview findings) | N/A |
| Zwerver 2011  Netherlands  [53] | Single cohort (interviews) | Depression | Disability assessment (insurance medical practice) (existing) | Insurance physicians | (Attitude – Social norm – self-Efficacy model) | N/A | The barriers or the support in the process of guidelines implementation can influence the change objectives for the environment. For this reason, a theoretical model that describes behavior was looked at. The ASE model describes how a person’s attitude, social influence, and self-efficacy influence behavior (describes implementation or its determinants) | Type 2: The ASE model was used as theoretical basis for assessing interview findings and selecting interventions using the Intervention Mapping process (select and tailor intervention) | Based on Intervention Mapping, proposed educational meetings, educational material, audit and feedback, ongoing training |
| Study | Research design | Health care issue | Guideline topic (new or existing) | Physician specialty | Theory (model or framework) | Type of theory | Rationale for theory (rationale category) | Type of study and Use of theory | Type of intervention planned or evaluated |
| Askelson  2010  United States  [54] | Single cohort study (questionnaire) | HPV | Vaccination of girls against HPV (prevention) (existing) | General practice physicians, family practice physicians, and pediatricians | TPB | Classic | The TPB has the ability to predict the intention to perform a behavior (validated, describes implementation or its determinants) | Type 1: The TPB constructs were measured in the questionnaire (inform survey questions) | N/A |
| Bekkers 2010  UK  [55] | Qualitative study embedded in a randomized controlled trial | Antibiotic resistance | Prescribing, management of sore throat (existing) | General practitioners, nurses | TPB, social learning theory | Classic | Theories “address both the how and why of change” (describes implementation or its determinants) | Type 3: Theory was used to inform intervention design: “The why of change is anchored in exposure to evidence and expert opinion…the how of change is captured in the detailed presentation of clear communication strategies that should enable clinicians to assess patients’ unvoiced agendas and identify and respond to information needs” | Clinical intervention: reduced antibiotic prescribing  Behavioural intervention: online educational modules and materials, in-person interactive workshop led by trained facilitator, educational videos, self-report of experiences by participants, web forum |
| Van Den Boogaard 2010 Netherlands  [56] | Single cohort (interviews) | Recurrent miscarriage | Guidelines on RM practice (existing) | Gynecologists, residents in obstetrics and gynecology, fertility doctors, resident geneticists | (Cabana framework of barriers to physician guideline adherence) | N/A | Both models categorize barriers and facilitators according to 4 similar domains (describes implementation or its determinants) | Type 1: Models were used to structure the interviews (inform interview questions) | N/A |
| Waddimba 2010 United States  [57] | Single cohort (questionnaire) | Asthma, diabetes, otitis media, and sinusitis | Care guidelines for asthma, diabetes, otitis media, and sinusitis (existing) | Family practitioners, internists, pediatricians | TPB (adapted) | Classic | The TPB states that work performance is directly predicted by behavioral intentions, which are in turn determined by attitudinal factors. In health care, the link between attitudinal factors and providers’ clinical behavior is supported by the literature (validated, describes implementation or its determinants) | Type 1: Items in the questionnaire assessed domains of the TPB (inform survey questions) | N/A |
| Study | Research design | Health care issue | Guideline topic (new or existing) | Physician specialty | Theory (model or framework) | Type of theory | Rationale for theory (rationale category) | Type of study and Use of theory | Type of intervention planned or evaluated |
| Bartholomew 2009 United States  [58] | Single cohort (before-after) | High blood pressure | Prevention, detection, evaluation, and treatment of high blood pressure (new) | Health care providers who manage hypertension | Social cognitive theory | Classic | SCT was chosen because it focuses on both individual cognitive influences on behavior and on social environmental influences. It provides a framework that incorporates categories of barriers and facilitators of the adoption of evidence based practice changes (describes implementation or its determinants) | Type 3: SCT was used to identify hypothetical barriers and facilitators which informed the selection and design of interventions recommended by the research team based on systematic Intervention Mapping process | Clinical intervention: anti-hypertensive therapy with diuretic  Behavioural intervention: academic detailing by trained educators plus a variety of educational materials, mass media patient messages to stimulate communication with physicians, and requests to pharmacy benefit systems and professional associations to appeal to physicians |
| Rashidian 2009 Iran  [59] | Single cohort (interview) | Asthma, coronary heart disease, depression, epilepsy, menorrhagia | Asthma treatment, coronary heart disease prevention, depression treatment, epilepsy treatment, prescribing for menorrhagia (existing) | General practitioners (who have experience of guideline implementation or development, or have experience evaluating clinical guidelines) | TPB | Classic | Limited applications had suggested that the TPB was useful in understanding provider behavior (used by others, describes implementation or its determinants) | Type 1: The TPB was used to design the study and framework method for the analysis (analyze interview findings) | N/A |

| Study | Research design | Health care issue | Guideline topic (new or existing) | Physician specialty | Theory (model or framework) | Type of theory | Rationale for theory (rationale category) | Type of study and Use of theory | | Type of intervention planned or evaluated | |
| --- | --- | --- | --- | --- | --- | --- | --- | --- | --- | --- | --- |
| Vogt  2009  UK  [60] | Randomized controlled trial | Smoking | Smoking cessation (existing) | General practitioners | Social cognitive theory, (elaboration likelihood model), self-perception theory | Classic (N/A for model) | One of the most influential motivational theories or behavior is social cognitive theory (used by others, validated). The elaboration likelihood model has been used to change a wide range of behaviors (used by others, validated). Self-perception theory has also been used to influence behavior (used by others, validated) | Type 3: Each theory or model informed the selection and design of a different trial arm intervention. SCT was related to information provision about cost-effectiveness; ELM is based on reinforcing information; SPT is based on providing feedback on past behaviour | | Clinical intervention: smoking cessation counselling  Behavioural intervention: arm 1 – cost effectiveness information in the form of absolute quit rate and number needed to treat; arm 2 – multiple choice quiz with answers; arm 3 – self-reported rates of smoking cessation counselling | |
| Althabe 2008 Argentina  [61] | Randomized controlled trial | Vaginal delivery | Episiotomy and management of the third stage of labor (new) | Birth attendants (physicians, residents, or midwives) | Diffusion of innovation theory | Classic | NR | Type 3: NR | | Clinical intervention: use of prophylactic oxytocin and episiotomy  Behavioural intervention: opinion leaders were trained at a workshop to develop a guideline and another workshop to develop training skills. At their hospital they disseminated guidelines and educational materials, trained staff, shared reminders, and generated monthly reports on rates of use of episiotomy and oxytocin | |
| Green 2008 UK  [62] | Single cohort (questionnaire) | Eating disorders (AN and BN) | Treatment of anorexia nervosa and bulimia nervosa (referral) (existing) | General practitioners | TPB | Classic | The TPB states that attitudes towards the behavior, subjective norms, and perceived behavioral control predict behavioral intention, which is linked to behavior (describes implementation or its determinants) | Type 1: The TPB was used to look at how GPs responded to patient vignettes (analyze survey findings) | | N/A | |
| Study | Research design | Health care issue | Guideline topic (new or existing) | Physician specialty | Theory (model or framework) | Type of theory | Rationale for theory (rationale category) | | Type of study and Use of theory | | Type of intervention planned or evaluated |
| McGinty 2008 United States  [63] | Single cohort (questionnaire) | Acute myocardial infarction | Treatment for acute myocardial infarction (existing) | Noninterventional cardiologists, interventional cardiologists, hospitalist/ intensivists | TPB | Classic | NR | | Type 1: Aspects of the TPB was used in the survey (informed survey questions) | | N/A |
| Griffiths 2007 UK  [64] | Randomized controlled trial | Tuberculosis | Tuberculosis screening (existing) | General practitioners and practice nurses | (Social influence model of behavior change) | N/A | NR | | Type 3: The design of educational outreach visits was guided by SI model; a specialist nurse and local GP discussed guidelines, provided training in tuberculin skin testing and modeled this behaviour, provided educational materials, and followed up by phone to reinforce the message and address issues. | | Clinical intervention: verbal tuberculosis screening and tuberculin skin testing  Behavioural intervention:  educational outreach, computerized screening prompts, tuberculin skin test equipment, financial incentive for every skin test completed |
| Luxford 2006 Australia  [65] | Single cohort (questionnaire) | Cancer | Management of colorectal cancer (new) | Colorectal surgeons, oncologists, general surgeons, gastroenterologists, policy makers, psychologists | Diffusion of innovation theory | Classic | The theory of diffusion of innovations has been acknowledged as useful for considering the adoption of new clinical practices (validated, used by others) | | Type 1: Theory was used to develop a matrix tool to probe for determinants of guideline use (informed survey questions) | | N/A |
| Rebergen  2006  Netherlands  [66] | Single cohort (questionnaire) | Mental health | Management of employees with mental health problems (existing) | Occupational physicians | TPB | Classic | In implementation research, guideline adherence has been examined using the TPB, which predicts the occurrence of a specific behavior provided that the behavior is intentional (used by others, describes implementation or its determinants) | | Type 1: A questionnaire was developed using the TPB (informed survey questions) | | N/A |

| Study | Research design | Health care issue | Guideline topic (new or existing) | Physician specialty | Theory (model or framework) | Type of theory | Rationale for theory (rationale category) | Type of study and Use of theory | Type of intervention planned or evaluated |
| --- | --- | --- | --- | --- | --- | --- | --- | --- | --- |
| Reyna 2006 United States  [67] | Single cohort (questionnaire) | Unstable angina | Guidelines for unstable angina (existing) | Family practice, emergency medicine, internal medicine, cardiology | Fuzzy-trace theory | Classic | According to fuzzy-trace theory, as people become more knowledgeable and experienced at a task, their information processing becomes more gist-based. It also predicts that more knowledgeable decision makers should be able to discriminate better among categories (describes implementation or its determinants) | Type 1: Fuzzy trace theory was used for analysis (analyze survey findings) | N/A |

TDF, Theoretical domains framework; TPB, Theory of Planned Behaviour; NPT, Normalization Process Theory. N/A not applicable; NR not reported
