# Supplementary material for: Use of theory to plan or evaluate guideline implementation among physicians: a scoping review
Source: Implement Sci. 2017 Feb 27;12:26. doi: 10.1186/s13012-017-0557-0 (PMC5327520; doi:10.1186/s13012-017-0557-0)
Supplement: Additional file 3: — Description of interventions evaluated in included studies. (DOC 58 kb) [file 13012_2017_557_MOESM3_ESM.doc]

Additional File 3. Description of interventions evaluated in included studies

| Study  Design  Intervention  Theory | Participants, Setting | Characteristics of those delivering the intervention | Intervention content | Mode of delivery | Intensity, Duration | Main Findings |
| --- | --- | --- | --- | --- | --- | --- |
| Ong 2014 UK  [32]  Qualitative study embedded in an RCT  Multifaceted  Normalization Process Theory | Interviews were held with 10 GPs from 8 practices in the West Midlands and North West of England taking part in an RCT of the intervention | GP facilitators delivered the intervention workshops | Each of the 4 workshops used a mix of didactic, interactive and role play components to convey clinical information about OA and its management, and use of the NICE OA guideline, OA guidebook for patients, and OA consultation model. Participants also received a computer template that prompted GPs to ask six questions of patients who presented with joint pain | Workshops were delivered in general practices | The intervention was delivered in 4 sessions, lasting 1 or 2 hours each, about 2-3 weeks apart from August 2011 to July 2012 | Interviews were used to assess fidelity of implementation of the intervention.  Barriers: Limited time available within the consultation, not patient friendly (thought by some)  Outcomes: GPs noted several benefits (does not require too much extra time, contributes to continuing education credits, addresses a common condition seen in their practices) and thought that, being led by GPs, it was trustworthy. After training, all GPs except one emphasised that their thinking changed about osteoarthritis. They felt that the NICE guidelines were more applicable as they were translated into a "toolbox". Most GPs were comfortable with the new intervention (giving patients a guidebook and referring patients to nurses for support). |
| Duff 2013 Australia  [39]  Single cohort before/after  Multifaceted  Social Marketing Theory | 15 male and 4 female physicians (median age 54 years) at an acute care private hospital in Sydney, Australia, who admitted at least 40 medical (non-surgical) patients annually, including cardiologists, neurologists, nephrologists, medical oncologists, immunologists, rheumatologists, gastroenterologists, and thoracic physicians. 300 consecutive patients were audited before (n=150) and after (n=150) the intervention period. | A peer facilitator, a vascular medicine physician with expertise in venous thromboembolism (VTE) prevention, received a two-day training workshop in social marketing and persuasive communication. | Educational outreach visits (EOV) on the provision of VTE prophylaxis to hospitalized medical patients. The EOV content was collaboratively developed by a multidisciplinary team of healthcare professionals and based on social marketing theory. Messages: VTE results in significant mortality, morbidity and resource expenditure; patients must have their risks assessed including clotting, bleeding risk, and contraindications to VTE prophylaxis; patients must receive appropriate prophylaxis based on their risk assessment; patients must be monitored for signs of VTE or other adverse events. | The facilitator visited each consenting participant in their office or clinical areas. The message was delivered verbally along with a graphic educational resource. | 2 trial visits, 1 intervention period of 2 months. The average length of each EOV was 15 minutes (IQ range 15 to 20) and the average time spent arranging and conducting each visit was 92 minutes (IQ range 78 to 129). | Outcomes: Proportion of medical patients receiving VTE prophylaxis improved significantly from baseline (54% pre-intervention, 70% post-intervention, 95% CI 5 to 26, p=0.004); 16 physicians (84%) reported that the intervention was effective or extremely effective at increasing their knowledge about VTE prophylaxis, 78% gave a verbal commitment to provide evidence-based prophylaxis. |

| Study  Design  Intervention  Theory | Participants, Setting | Characteristics of those delivering the intervention | Intervention content | Mode of delivery | Intensity, Duration | Main Findings |
| --- | --- | --- | --- | --- | --- | --- |
| French 2013 Australia  [40]  RCT  Single  Theoretical Domains Framework, Theory of Planned Behaviour | All 1688 general practices were approached in Victoria, Australia (4978 GPs). 92 general practices were randomized. 45 general practices (59 GPs) were allocated to intervention and 47 general practices (53 GPs) to control. At least 38 general practices (48 GPs) were available for analysis from the intervention group, and 40 general practices (44 GPs) from the control group. 36/59 GPs (61%) in the intervention group attended the two facilitated workshops. 29 patient participants were recruited | A “peer expert” (a trained GP facilitator) | The intervention group received an intervention delivered via two facilitated, interactive, educational workshops (didactic lectures, small group discussions and activities), informed by the TDF and the TPB, and tailored to barriers and facilitators that had been identified in focus group interviews. The intervention comprised a combination of behaviour change techniques, including providing instruction; modelling/demonstrating the behaviour by a peer expert; providing information on consequences; prompt barrier identification; time management; prompt specific goal setting; rehearsal; persuasive communication; and scripting. A DVD, with film footage of the workshops and electronic resources, was produced to distribute to all GPs in the intervention group, intended primarily for those who could not attend the workshops. The control group received a printed copy of the guideline and information on how to access an electronic version of the guideline, in accordance with existing dissemination policy. | Recruitment was done by regular mail. A random selection of GPs was also contacted by telephone | Two workshops were held, each of 3 hours’ duration. There are two workshops. The workshops were held between June and September 2007. | Outcomes: A questionnaire was distributed to GPs 12 months after intervention/control delivery and contained 51 items to measure behavioral constructs (2 guideline recommended behaviors) and 4 patient vignettes to simulate clinical decision making about acute LBP management in specific situations. GP plain film x-ray and CT scan referral were measured via medical administrative data. For the behavioural construct outcomes, the intervention effects, while favoring the intervention, were typically small. GPs in the intervention group had greater intentions to practice consistent with the guideline recommendation of x-ray referral. For the behavioral simulation outcomes, at 12 months post-intervention, GPs in the intervention group had larger odds of adhering to the guideline recommendations about x-ray and imaging (OR 1.76, 95%CI 1.01, 3.05), and to give advice to stay active as measured in response to vignettes (OR 4.49, 95%CI 1.90 to 10.60). For administrative data outcomes, there was no statistically significant difference in referrals |

| Study  Design  Intervention  Theory | Participants, Setting | Characteristics of those delivering the intervention | Intervention content | Mode of delivery | Intensity, Duration | Main Findings |
| --- | --- | --- | --- | --- | --- | --- |
| Bekkers 2010  UK  [55]  Qualitative study embedded in an RCT  Multifaceted  Theory of Planned Behaviour, Social Learning Theory | 30 GPs were interviewed from 33 intervention group general practices taking part in a trial to reduce antibiotic prescribing | Part 3 of the intervention was facilitated by an educator. The other parts were delivered online. | The educational program was comprised of 5 parts, supplemented with an ongoing web forum (part 6) and an online booster session (part 7). Parts 1 and 2 involve an online introduction to antibiotic resistance and prescribing, and provided case scenarios for reflection and examples of the latest evidence. Part 3 was an on-site, face-to-face seminar (discussion about practice-unique prescribing data, and resistance data measured from samples submitted by the practice over a 5-10 year period prior to the study). Part 4 used video scenarios to demonstrate key consultation strategies. Part 5 consolidated the learning experience by asking clinicians to describe and reflect on 3 examples from their own clinical practice. | Online interactive modules and discussion plus in-person didactic and interactive seminar | Duration of the session NR. Booster provided around 6 months after completion of core program. | Outcomes: Reported increased awareness of antibiotic resistance, greater self-confidence in reducing antibiotic prescribing, change in consultation style and antibiotic prescribing behaviour.  Reported practice changes included adopting a practice-wide policy of antibiotic prescription reduction. The components of the intervention described as having had the greatest influence on changing clinician behaviour were the up-to-date research evidence resources, simple and effective communication skills presented in on-line videos, and presentation of the practice's own antibiotic prescribing levels combined with an overview of local resistance data. Some interviewees considered the research evidence not directly relevant to their own clinical practice, or found it too difficult to process online. The web forum was dismissed by many as irrelevant |
| Bartholomew 2009 United States  [58]  Single cohort before/after  Multifaceted  Social Cognitive Theory | Educators delivered 1,698 outreach presentations to 18,524 clinicians in 41 states. Questionnaires were completed by a convenience sample of 1,709 respondents for the pre-test and 1,617 respondents for the post-test. Information was mailed to 55 individuals at 20 professional, 8 formulary systems, 14 health plans or insurance companies that provide web-based information to patients and 74 mass media outlets | Investigator educators (mostly physicians who had participated in the ALLHAT trial as clinical investigators) were trained to conduct outreach presentations | Plans for program delivery were based on social cognitive theory. Educators conducted academic detailing including discussion of recommendations for managing hypertension with diuretics and lifestyle modification. They also provided print material including a newsletter, pocket cards, ALLHAT trial publications, tips for accurate blood pressure measurement, a booklet on dietary management of hypertension, exam room posters and prescription pads. Professionals associations were provided with template letters and newsletter articles to be used when communicating with members. Formularies were provided with ALLHAT trial publications, the prescribing recommendations, and information about formulary management and costs. A press release about the project was released to mass media outlets | Investigator educators | Presentations between September 2004 and March 2007 | Outcomes: Based on the questionnaire, outcome expectations and intent to prescribe diuretics increased significantly between pre- and post-test (p<0.005) |

| Study  Design  Intervention  Theory | Participants, Setting | Characteristics of those delivering the intervention | Intervention content | Mode of delivery | Intensity, Duration | Main Findings |
| --- | --- | --- | --- | --- | --- | --- |
| Vogt 2009 UK  [60]  RCT  Single (in each of three trial arms)  Social Cognitive Theory, Self-Perception Theory, Elaboration Likelihood Model | All participants were general practitioners practicing in the UK and users of a free Internet-based medical information service provider. Of the 251 PCPs who completed the baseline questionnaire, 210 (83%) completed the post intervention questionnaire and 182 (71%) completed all 3 questionnaires | NR | The social cognitive theory group was given information about the effectiveness and cost effectiveness of smoking cessation services. The SCT+elaboration likelihood model group was given the same information, but it was provided in answers to a quiz. The self-perception theory group was first provided with positive information about the rate at which they helped smokers and information with the aim of recommending smoking cessation services. The control group received information about the history of smoking cessation services in the UK. Behavior, intention, and outcome expectations were assessed at baseline and post intervention | Email | PCPs that accessed the information service provider during 3 days in July 2005 were invited into the study. 3 days after PCPs completed the baseline questionnaire, they received an email with their allocated intervention. Immediately after exposure to this intervention, they were presented with the post intervention questionnaire (T1). 7 days later, PCPs received an email with a link to the post intervention questionnaire (T2). Both emails informed PCPs that they had 2 days to access the link | Outcomes: None of the means differed between the SPT and the control conditions. The means of expectations about effectiveness (B=0.47, SE=0.21, p<0.05) and cost effectiveness (B=0.63, SE=0.26, p<0.01) of smoking cessation services in the SCT+ELM condition were higher than in the control condition. The means of expectations about effectiveness (B=0.49, SE=0.21, P<0.05) and cost effectiveness (B=0.94, SE=0.26, P<0.01), and behavior (B=1.09, SE=0.46, P<0.05) in the SCT condition were higher than those in the control condition. The SCT condition recommended 1.1 more smokers per week to smoking cessation services than did PCPs in the control condition. The differences in behavior between the SCT condition and the control condition remained (B=0.96, SE=0.46, p<0.05), even though intentions did not differ |

| Study  Design  Intervention  Theory | Participants, Setting | Characteristics of those delivering the intervention | Intervention content | Mode of delivery | Intensity, Duration | Main Findings |
| --- | --- | --- | --- | --- | --- | --- |
| Althabe 2008 Argentina  [61]  RCT  Multifaceted  Diffusion of Innovation Theory | The trial was conducted in 19 public maternity hospitals in Argentina and Uruguay. The readiness to change survey was administered preintervention to 593 birth attendants (response rate=89.3% in intervention hospital and 82.6% in control) and postintervention to 617 birth attendants (mean response rate was 90.5% in intervention hospitals and 82.1% in control hospitals). Participants included midwives, obstetrician-gynecologists and resident physicians. | Teams of three to six birth attendants were identified as opinion leaders with the use of a previously validated questionnaire | Intervention based on the conceptual framework of diffusion theory. The opinion leaders disseminated the guidelines, trained and visited birth attendants, and developed reminders. The teams also produced monthly reports on rates of use of episiotomy and prophylactic oxytocin based on hospital clinical data. The birth attendants completed anonymous, self-administered questionnaires before and after intervention to describe their readiness to adopt or maintain active management of the third stage of labor or to adopt the selective use of episiotomy | Opinion leaders delivered guidelines, provided training, and generated and shared monthly performance reports at participating sites | 18 month intervention (Sept 2003-Dec 2005). Data were collected during a 3 month period before randomization (baseline), the last 3 months of the 18 month intervention (post intervention), and the 3 months beginning 1 year after the beginning of the post intervention interval (follow-up) | Outcomes: Results of post-training surveys showed that the opinion leaders were nearly unanimous in support of the recommendations. Compliance with the intervention was high. The rate of prophylactic use of oxytocin increased from 2.1% to 83.6% in the intervention hospitals and 2.6% to 12.3% in control. Absolute change of 67.5% (P=0.01). The intervention was associated with a decrease in the median episiotomy rate in intervention hospitals from 41.1% at baseline to 29.9% at the end of the intervention. The rate was stable in control groups. The difference in rate change was -10.9% (P<0.001). In the intervention hospitals, the median rate of readiness to change increased from 14.4% to 55.9%, whereas the rates in the control hospitals remained stable. The absolute difference between the rate changes was 38.4% (P<0.001). Those practices remained stable at the 1 year follow-up |
| Griffiths 2007 UK  [64]  RCT  Multifaceted  Social Influence Model of Behavior Change | 52 primary care practices from one district; 44,986 and 48,984 patients were registered from intervention and control practices, respectively | A tuberculosis specialist nurse and an academic GP conducted outreach visits | The interventions consisted of an educational outreach visit to each intervention practice, computer prompts to remind clinicians to ask screening questions, equipment for tuberculin skin testing, telephone support by a tuberculosis specialist nurse for advice, and a financial incentive of $12.9 for every tuberculin skin test. The outreach visit used the social influence model of behavior change | In person outreach visit, telephone follow-up, computer reminders and financial incentive for each test performed | Trial took place from June 1, 2002 to Oct 1, 204 | Outcomes: Compared with control, intervention practices had higher rates of screening (57% vs 0.4% of patients), diagnosis of active (47% vs 34%, OR 1.68, p=0.03) and latent tuberculosis (19% vs 9%, p=0.06) and use of vaccine (26.8/1000 vs 3.8/1000, p<0.001) |

NR not reported; TDF, Theoretical Domains Framework; TPB, Theory of Planned Behaviour
